# Aged Opossums Show Alterations in Spatial Learning Behavior and Reduced Neurogenesis in the Dentate Gyrus

**DOI:** 10.3389/fnins.2019.01210

**Published:** 2019-11-12

**Authors:** Beata Tepper, Agata Aniszewska, Katarzyna Bartkowska, Lilianna Grochocka, Krzysztof Turlejski, Ruzanna Djavadian

**Affiliations:** ^1^Nencki Institute of Experimental Biology, Polish Academy of Sciences, Warsaw, Poland; ^2^Department of Public Health and Caring Sciences, Uppsala University, Uppsala, Sweden; ^3^Faculty of Biology and Environmental Sciences, Cardinal Stefan Wyszyński University in Warsaw, Warsaw, Poland

**Keywords:** adult neurogenesis, dentate gyrus, learning and memory, doublecortin, water maze

## Abstract

In many mammalian species including opossums, adult neurogenesis, the function of which is not completely understood, declines with aging. Aging also causes impairment of cognition. To understand whether new neurons contribute to learning and memory, we performed experiments on young and aged laboratory opossums, *Monodelphis domestica*, and examined the association between spatial memory using the Morris water maze test and the rate of adult neurogenesis in the dentate gyrus (DG). Modification of this test allowed us to assess how both young and aged opossums learn and remember the location of the platform in the water maze. We found that both young and aged opossums were motivated to perform this task. However, aged opossums needed more time to achieve the test than young opossums. Classical parameters measuring spatial learning in a water maze during a probe test showed that young opossums spent more time in the platform zone crossing it more often than aged opossums. Additionally, hippocampal neurogenesis was lower in the aged opossums than in the young animals but new neurons were still generated in the DG of aged opossums. Our data revealed individual differences in the levels of doublecortin in relation to memory performance across aged opossums. These differences were correlated with distinct behaviors, particularly, aged opossums with high levels of DCX achieved high performance levels in the water maze task. We, therefore suggest that new neurons in the DG of Monodelphis opossums contribute to learning and memory.

## Introduction

Adult neurogenesis occurs in two neurogenic areas of the mammalian brain: the subventricular zone (SVZ) of the lateral ventricles and the dentate gyrus (DG) of the hippocampal formation. The vast majority of newborn neurons in the SVZ migrate long distances to reach the olfactory bulb (OB), and differentiate into granule or periglomerular cells, whereas stem cells divide in the subgranular zone of the DG and migrate to the granular layer (for review, see Lledo and Valley, 2016; Obernier and Alvarez-Buylla, 2019). These adult-born neurons are stably incorporated into the hippocampal circuitry and function like other granular neurons of the DG (for review, see Gonçalves et al., 2016). In early stages of development neurons, derived from both neurogenic regions in the adult brain express doublecortin (DCX), known as a marker for immature neurons. DCX-expressing cells are not restricted to two neurogenic regions, but are also observed in the piriform cortex, the corpus callosum, the striatum and the amygdala (Nacher et al., 2001; Dayer et al., 2005; Zhang et al., 2009; Bartkowska et al., 2010).

Adult neurogenesis is conserved among mammalian species. However, a number of papers suggest that adult neurogenesis in humans is unique (for review, see Bergmann et al., 2015). First, the number of newly generated cells in the SVZ is drastically reduced after childbirth and is seen in up to 6–8 months old infants (Sanai et al., 2011; Wang et al., 2011). Apart from this, newly generated cells in the SVZ do not migrate to the OB but are added to the striatum (Ernst et al., 2014) and the piriform cortex (Klempin et al., 2011). However, some studies have reported opposite results. Progenitor cells labeled with progenitor markers and early neuronal markers have been shown in the OB of young and aged humans (Bédard and Parent, 2004; Curtis et al., 2007).

The rate of adult neurogenesis declines with age, or even ceases in some species such as Sorex shrews and in echolocating microbats (Amrein et al., 2007; Bartkowska et al., 2008). In humans, there are controversial data concerning adult neurogenesis in the DG. Some results show that the rate of neurogenesis in middle-aged humans and mice is comparable, whereas other studies report that new neurons proliferate only in the first year of infant life and the rate of adult-born neurons is very low in humans between 7 and 13 years of age (Spalding et al., 2013; Sorrells et al., 2018). On the other hand, newborn neurons have been reported in the DG of older humans (Boldrini et al., 2018). These discrepancies have been outlined in a recently published review by Kempermann et al. (2018), where a detailed overview of adult neurogenesis in the human DG is provided. They conclude that additional experiments, including single-cell RNA sequencing, need to be performed for a better understanding of adult neurogenesis.

Our previous work showed that neurogenesis is present throughout the lifespan in the DG of the opossum, *Monodelphis domestica*; however, its rate is reduced by half in old age (Grabiec et al., 2009). Additionally, we hypothesized that newborn neurons may be required for behavioral responses of adult opossums.

Here, we performed the Morris water maze test to test whether adult neurogenesis is required for behavioral response, specifically for spatial hippocampal memory. Next, using progenitor cell markers, we examined adult neurogenesis in young and aged animals, with particular reference to DCX-immunopositive cells.

## Materials and Methods

### Animals

Opossums breeding at the Nencki Institute of Experimental Biology colony were divided into two groups, young with an average age of 6.5 months and aged with an average age of 21.5 months. Since opossums are solitary animals, they were housed individually starting at 4 months of age. Each opossum’s cage was equipped with a plastic nest house.

Ecology of opossums is different from the rodents commonly used in the laboratory. Opossums live in savanna or grassland of South America, where the average climate is very mild. For this reason, the temperature in the Animal Facility of the Nencki Institute for opossums was set at 26°C, higher than for the laboratory rodents. The animals were kept under a 14/10-h light/dark cycle with self-regulated access to food and water.

All efforts were taken to minimize the number of animals used and the level of stress they endured. The experimental procedures complied with the Polish Law on Experiments on Animals, which implements the European Council Directive, and were approved by the Local Ethics Committee in Warsaw.

### Water Maze Test

The Morris water maze test was used to study spatial memory in young (*n* = 14) and aged (*n* = 14) opossums (Morris, 1981). The test apparatus was made of a white plastic pool of 1.5 m diameter and 0.6 m height, and filled with water at 26–27°C. The surface of the pool was virtually divided into four quadrants (I, II, III, and IV). A plastic platform with 10 cm diameter and 31 cm height was placed in the center of the quadrant II and water level in the pool was 2 cm above the platform. The opossum nest house was attached to the edge of the pool as a visual clue for the animals. On the wall within each quadrant, there was a marked starting place (NE, SE, SW, or NW). Some objects such as curtains, a hanger and a sink also served as a visual clue.

The experiment lasted 6 days, including 5 days of training (learning days) and a probe test on the sixth day. On the first day of training, the opossums were first released for 30 s in the pool and then transferred to the platform from which they were removed after 60 s. The opossums were given 4 trials. The first trial began with the starting point in the NE quadrant I, and for each consecutive trial, the starting point was changed in a clockwise direction from the second to the fourth quadrant. For the remaining 4 consecutive days of training, each day the starting point (first trial) moved to the next quadrant in relation to the previous day. For the remaining trials, the starting point was changed in a clockwise direction. Each trial lasted 60 s. If the animal located the platform within 60 s, it spent 30 s on the platform and was later transferred to the cage. If the animal failed to reach the platform within 60 s, it was placed on the platform for 60 s and transferred to the cage. The opossums stayed in the cage for 3 min between trials.

On the sixth day of the experiment, 24 h after the last acquisition trial a probe test was administered. The animal was first placed for 60 s on the platform to demonstrate that the environment had not changed. Next, the platform was removed, and the opossum was allowed to swim for 60 s in the pool during which time spent in the platform zone and the number of platform zone crossings were measured. All other parameters evaluated during the learning test also were analyzed. The starting point for probe test was SE.

The experiment was recorded by a camera placed over the pool and analyzed by the EthoVision XT video tracking software (Noldus Information Technology). The frequency of swimming to the NE quadrant and the time to enter the platform were analyzed. The total distance and the swimming speed were also estimated. Additionally, thigmotaxis behavior was studied by analyzing thigmotactic responses, which were calculated as the duration of swimming in a circular zone of 10 cm along the pool wall. Since the platform was removed from the pool on the last day, the same parameters were measured except those for the platform itself.

### Animal Treatment and Tissue Preparation

Three 6-month-old and three 21-month-old aged opossums were injected twice with 75 mg/kg bromodeoxyuridine (BrdU, Sigma-Aldrich) at a 2 h interval. Four weeks after BrdU-injections opossums were perfused with saline followed by 4% paraformaldehyde in 0.1 M phosphate buffer (pH 7.4). Ten young and 10 aged opossums used for memory testing in the water maze test were also perfused. Their brains were removed, postfixed in the 4% paraformaldehyde solution, and cut into 40 μm coronal sections in a cryostat. The brain sections were arranged in a series of ten.

Four young and four aged opossums were euthanized by an injection of Morbital (200 mg/kg) after being tested in the Morris water maze test. Their brains were isolated and the different structures were separated on ice. The hippocampal formation involving mainly DG, the OB, and the cerebellum were collected and weighed separately. They were mechanically homogenized in lysis buffer with protease inhibitors (Roche), treated with detergents NP 40 (Fluka) and sodium dodecyl sulfate (SDS, Sigma), and were incubated for 15 min. Next, they were centrifuged at 14,000 rpm for 45 min at 4°C. The supernatant was collected, aliquoted, and stored at −70°C.

### Immunofluorescent Labeling

BrdU immunostaining was performed in a cohort of animals injected with BrdU, whereas DCX staining was performed on opossums that went through the water maze test.

Immunohistochemical staining was performed on free-floating sections. After 12 h incubation in saline-sodium citrate at 60°C, sections were denatured in 2 M HCl at 37°C for 30 min. To block endogenous peroxidases, the sections were soaked for 30 min in 3% H_2_O_2_ in Tris–buffered saline (TBS). The sections were then rinsed for 15 min in TBS-A (TBS with 0.1% Triton X-100), and 15 min in TBS-B (TBS-A with 0.05% BSA - bovine serum albumin, Sigma). After rinsing for 1 h in a 10% goat serum solution in TBS-B, the sections were incubated with a rat anti-BrdU monoclonal primary antibody (1:500, Santa Cruz) overnight at 4°C. After a 15-min wash with TBS-A and TBS-B, the sections were incubated for 60 min with a biotinylated goat secondary antibody (1:200, Jackson ImmunoResearch) in TBS-B. This was followed by washes with TBS-A and TBS-B with streptavidin conjugated with a fluorescent dye (1:500, Alexa Fluor 488; Molecular Probes) for 1 h.

Some brain sections were incubated for 1 h with either 10% normal goat serum (Millipore), or 10% normal chicken serum (Sigma-Aldrich) and 1% BSA in PBS. Next, the sections were incubated overnight in the goat anti-DCX (1:100, Santa Cruz Biotechnology), rabbit anti-NeuN (1:50, Cell Signaling), rabbit anti-Olig2 (1:100, Millipore), or mouse anti-GFAP (1:500, Sigma-Aldrich) primary antibodies. The appropriate secondary antibodies: goat anti-rabbit 568 (1:500, Abcam), chicken anti-goat 488 or goat anti-mouse 568 (1:500, AlexaFluor Invitrogene) were subsequently used. Some section after rinsing with PBS were counterstained with DAPI (1:5000, Sigma-Aldrich). Finally, the sections were mounted on slides and coverslipped with a 60% glycerol solution in PBS.

### Western Blot

The samples containing 50 μg of protein were loaded onto a 10% SDS polyacrylamide gel. Proteins were separated by SDS polyacrylamide gel electrophoresis (PAGE) method and were transferred to a nitrocellulose membrane (BioRad) by electrotransfer for 1.5 h at 300 mA. Next, the blots were blocked in 5% milk in Tris–buffered saline with Tween (0.2%) overnight at 4°C. The blots were incubated with mouse anti-DCX antibody (1:500, Santa Cruz) overnight at 4°C and after several washes, the goat anti-mouse IgG (1:2000, BioRad) secondary antibody was applied for 1 h. Finally, the enhanced chemiluminescence (ECL) reagent (Advansta) was applied to the blots.

GAPDH was used as a loading control. To visualize the GAPDH protein on the same blots, a Re-Blot Plus Stripping Solution (Millipore) was used for 15–20 min to remove the previously bound DXC protein. The blocking procedure was repeated as described above. The blots were incubated with a mouse anti-GAPDH (1:10000, Millipore) primary antibody and a goat anti-mouse IgG (1:7000, Chemicon) secondary antibody.

### Data Analysis and Statistics

The results obtained in behavioral tests during the training experiment were analyzed using two way repeated measures analysis of variance (ANOVA) followed by the *post hoc* Fisher’s least significance difference (LSD) analysis. Differences were considered significant for *p* < 0.05. Data from the probe day were analyzed using the Student’s *t*-test (two-tailed, two samples of unequal variance).

Western blots were scanned in a G:BOX Chemi XT4 equipped with a camera (Syngene) and images were analyzed using GeneSys (Syngene) software. The intensity of protein bands was determined by densitometric analysis. The Student’s *t*-test (two-tailed, two samples of unequal variance) was used for statistical analysis of protein concentrations and *p* < 0.05 was considered statistically significant.

The quantification method used for single and double immunolabeling was described by Grabiec et al. (2009). Images of immunostained sections were obtained with a fluorescence microscope (Nikon) and analyzed using the Neurolucida software (MBF Bioscience). For quantification of BrdU cells, each immunolabeled cell in a series of tenth brain section was counted with a x20 microscope objective, and the total number was multiplied by ten. Colocalization of two fluorescent labels was determined using a confocal laser microscope (Zeiss). In each brain 50 BrdU-labeled cells were counted with a x63 microscope objective, and percentages of colocalization of BrdU with NeuN, or BrdU with GFAP, or BrdU with Olig2 were calculated. The Student’s *t*-test or ANOVA was performed for statistical analysis of differences between groups. The number of DCX immunopositive cells was counted in 5 sections of the DG on both sides (ipsilateral and contralateral) using a confocal laser microscope (Zeiss) with a x20 objective. For each opossum, brain sections to be counted were randomly selected in a series of tenth. In all animals, the selected sections sampled both the anterior and the posterior parts of the DG. The relationship between the number of DCX immunopositive cells and memory performances in aged opossums was tested by the Pearson correlation test (two-tailed).

## Results

**FIGURE 1:**
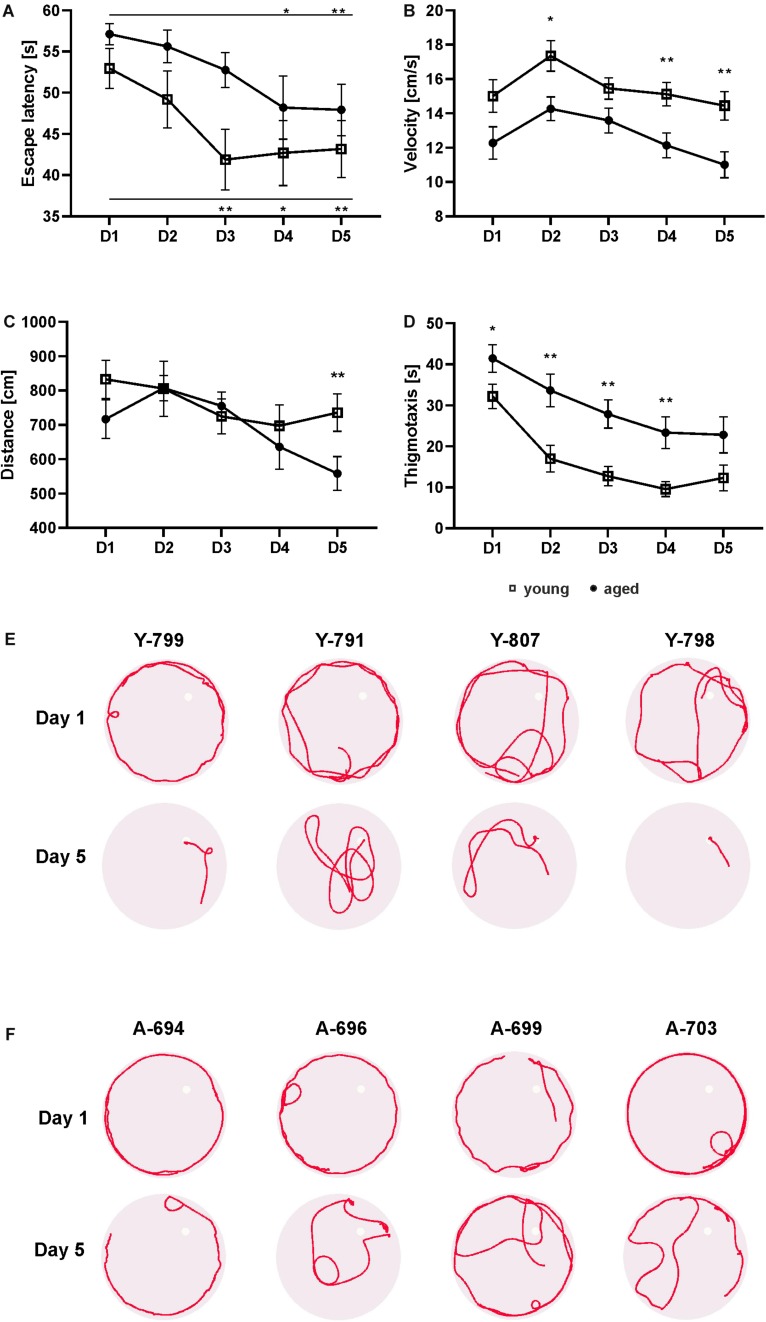
Behavior of young and aged opossums in the Morris water maze test during training. The young group consisted of 14 male opossums with an average age of 6.5 months, while the old group consisted of 14 opossums with an average age of 21.5 months, of which two were female. Both groups of opossums decreased the escape latency during five consecutive learning days **(A)**. The total distance traveled by young and aged opossums was the same during 4 days training, although on the last day the young opossums traveled a significantly longer distance than the aged opossum **(C)**. The swimming velocity **(B)** and the thigmotactic responses **(D)** were also different. In **(A–D)** squares refer to young opossums and circles refer to aged opossums. **(E,F)** Swimming path lengths for four young and four aged opossums on training day 1 and day 5. A small circle shows the placement of the hidden platform. The opossum number is at the top of each circle. Y, young; A, aged. ^∗^*p* < 0.05, ^∗∗^*p* < 0.01.

To assess whether animals learned and remembered the location of the hidden platform in the pool using visual cues, the escape latency was measured during five consecutive learning days (Figure 1A). Two way repeated measures ANOVA showed that although there was a significant effect of time [*F*(3.1, 80.9) = 5.89, *p* = 0.001] and of age [*F*(1, 26) = 4.75 *p* = 0.038] on latency, there was no significant interaction between these factors [*F*(4, 104) = 0.56, *p* = 0.694]. *Post hoc* Fisher’s LSD test identified a reduction in latency to reach the platform between the first day and the third day (*p* = 0.005), and subsequent days till the last day of training (*p* = 0.007) in the group of young possums (Figure 1A). The latency to the platform was gradually shortened in the aged group of opossums on the subsequent training days. On the first day of training, the aged animals reached the platform within an average of 57.09 ± 1.30 s whereas the latency on the last day was 47.93 ± 3.11 s (Figure 1A). The LSD test showed a significant difference starting from the fourth day (*p* = 0.026) in aged opossums.

The swimming velocity of aged opossums was significantly lower compared to young animals (Figure 1B). Two way repeated measures ANOVA showed a significant difference in velocity between groups [*F*(1, 26) = 14.59, *p* > 0.001] and time [*F*(3.2, 83.5) = 6.18, *p* > 0.001]. The average velocity of young animals was 15.47 ± 0.84 cm/s versus 12.65 ± 0.77 cm/s in the aged ones, which indicates that young opossums swam approximately 20% faster than the aged animals (Figure 1B).

We also recorded the total distance traveled by the opossum in the five learning days (Figure 1C). Although the distance traveled by the young and old animals did not differ significantly [*F*(1, 26) = 1.58, *p* = 0.22], the total distance traveled by both was reduced over the course of the training [*F*(3.5, 91.7) = 4.07, *p* = 0.006]. On the first day, the average distance traveled by an opossum was 774.85 ± 56.03 cm, while on the last day of training it was 647.1 ± 52.01 cm (Figure 1C). Interestingly, *post hoc* Fisher’s LSD revealed that on the last day the aged opossums traveled a significantly shorter distance (558.3 ± 49.18) than the young opossums (735.9 ± 54.85) (*p* = 0.023).

The thigmotaxis behavior, defined as the tendency of the animal to swim close to the pool wall, was observed as a stress parameter. A circular zone 10 cm from the pool wall was marked and the thigmotactic responses were estimated by the time spent in this zone, measured in seconds. We found a significant difference in the thigmotactic responses [*F*(1, 26) = 12.39, *p* = 0.001] and effect of time [*F*(2.6, 66.9) = 26.04, *p* < 0.0001]. Although on subsequent days of training, a gradual decrease in thigmotaxis was observed, for every day of training, aged opossums displayed more thigmotaxy than young opossums. Starting from the second day to the fifth day of training, the aged opossums spent twice the length of time in the thigmotactic area than young opossums (Figure 1D). Swimming path lengths as illustrated in Figures 1E,F clearly showed that on the first day both young and aged opossums were characterized with a quite similar performance in the water maze; almost all opossums swam mainly close the pool wall. Yet, on the last training day young opossums hardly displayed thigmotaxis behavior, while the majority of aged opossums exhibited swimming near the pool wall (Figures 1E,F).

### Behavior of Opossums in the Morris Water Maze Test: Probe Test

**FIGURE 2:**
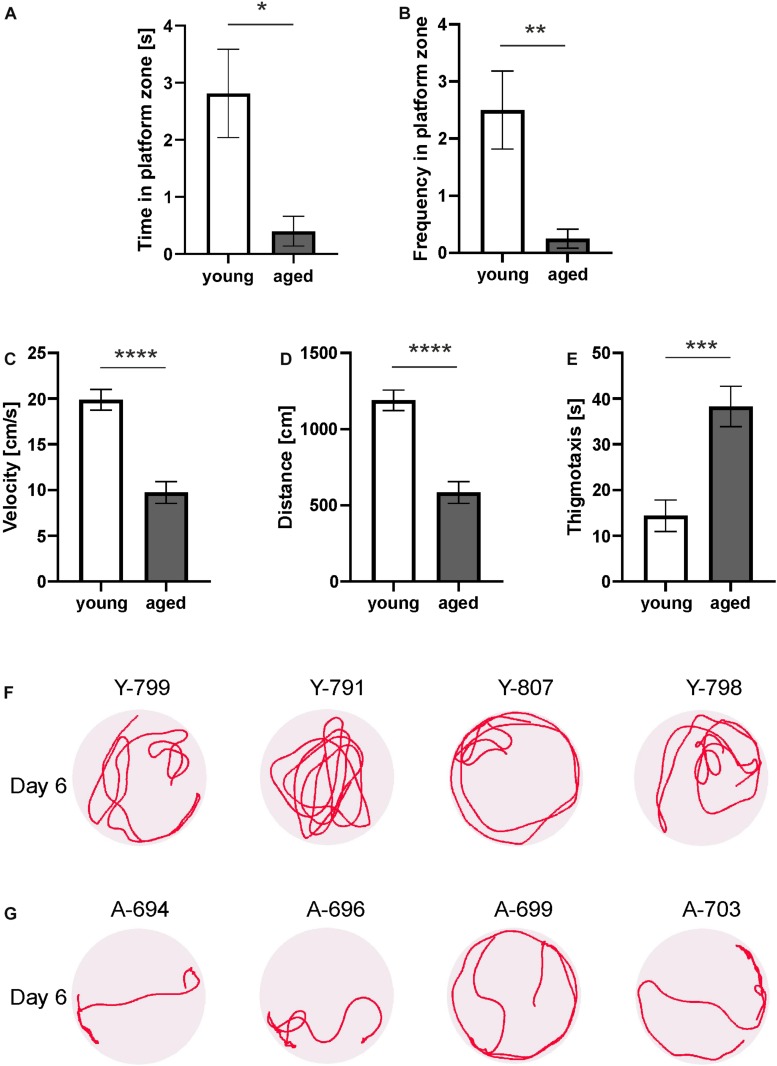
Behavior of young and aged opossums in the Morris water maze test during a probe test. **(A)** Time spent in the platform zone, **(B)** the number of the platform zone crossings, **(C)** velocity, **(D)** traveled distance, **(E)** time spent on thigmotaxis activity. **(F,G)** Swimming path lengths for four young and four aged opossums on the probe day. A small circle shows the placement of the hidden platform. The opossum number is at the top of each circle. Y, young; A, aged. ^∗^*p* < 0.05, ^∗∗^*p* < 0.01, ^∗∗∗^*p* < 0.001, ^∗∗∗∗^*p* < 0.0001.

To assess spatial memory on the sixth day, the opossums were administered a probe trial. In this probe trial both groups of opossums entered the target quadrant and spent approximately 1/4 of time there. However, young opossums spent more time in the platform zone and showed an increased number of platform crossings (Figures 2A,B). The *t*-test revealed that these differences were significant (*p* < 0.01).

Other parameters (velocity, distance, and thigmotaxis) measured on training days were also analyzed in the probe test. We observed that the velocity of the young and aged opossums during the probe test on the last day did not differ from that on the fifth day of training (Figure 2C). The swimming velocity of the young opossums was significantly higher than that of aged animals (*p* < 0.0001).

On the last day of the experiment, the young opossums traveled a greater distance than the old opossums (Figure 2D). The average distance covered in the probe test was 1189.4 ± 51.96 cm for young as opposed to 584.1 ± 55.02 cm for aged animals. Young animals displayed less thigmotactic responses and swam close to the pool wall one third of the time less as compared to aged opossums (Figure 2E).

We finally analyzed swimming path lengths and found that young and aged opossums displayed different strategy for achieving memory (Figures 2F,G). On the last day, when the platform was removed, young opossums swam mostly far from the pool walls (Figure 2F), while aged opossums spent most time in the thigmotactic area, next to the walls (Figure 2G).

### New Neurons in the Adult Brains of the Young and Aged of Opossums

**FIGURE 3:**
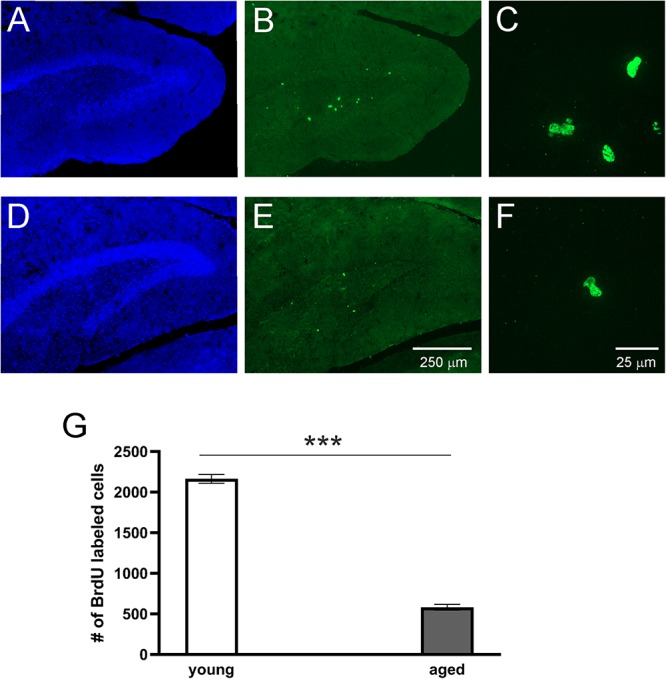
Proliferation of cells in the DG of adult opossums. Confocal images showing DAPI (blue) and BrdU (green) immunofluorescence stainings in the DG of young **(A–C)** and aged **(D–F)** opossums. **(C,F)** – High magnification from the images **(B)** and **(E)**, respectively. **(G)** The number of BrdU immunopositive cells in both groups of opossums. ^∗∗∗^*p* < 0.001.

**FIGURE 4:**
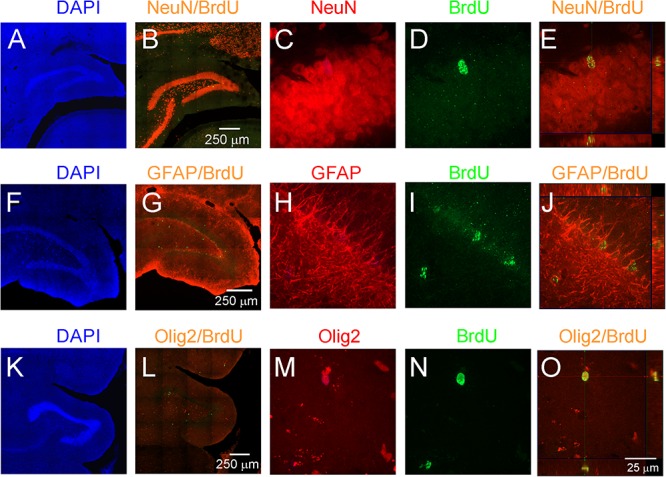
Proliferation and neurogenesis in the DG of adult opossums. **(A–E)** DAPI (blue), NeuN (red) and BrdU (green); **(F–J)** DAPI (blue), GFAP (red) and BrdU (green); **(K–O)** DAPI (blue), Olig 2 (red) and BrdU (green) double immunostaining in the DG. **(E,J,O)** Higher magnification of orthogonal confocal images for NeuN/BrdU, GFAP/BrdU and Olig2/BrdU, respectively. The scale bar in **(B)** refers to **(A,B)**. The scale bar in **(G)** refers to **(F,G)**. The scale bar in **(L)** refers to **(K,L)**. The scale bar in **(O)** refers to **(C–E)**, **(H–J)**, **(M–O)**.

To study adult neurogenesis a separate cohort of young and aged opossums was injected with BrdU. Four weeks later opossums were perfused and newly generated cells were determined by immunohistochemistry. BrdU labeled cells were observed in the DG of both young (Figures 3A–C) and aged (Figures 3D–F) opossums. However, in the DG of young opossums the number of BrdU labeled cells was about three times higher than in the aged opossums (Figure 3G). To define the type of newly generated cells in the DG different immunofluorescent markers, BrdU/NeuN, BrdU/GFAP or BrdU/Olig2 were used for immunostaining brain sections. Most of BrdU labeled cells (62%) located in the granule/subgranule cell layer of the DG expressed NeuN (Figures 4A–E). None of BrdU labeled cell located in the hilus of DG expressed NeuN, while 20% of BrdU positive cells in the hilus of the DG showed simultaneously expression of Olig2, which is an oligodendrocyte specific protein (Figures 4K–O). In turn, 14% of BrdU labeled cells in the DG had the astrocyte phenotype, expressing GFAP (Figures 4F–J).

**FIGURE 5:**
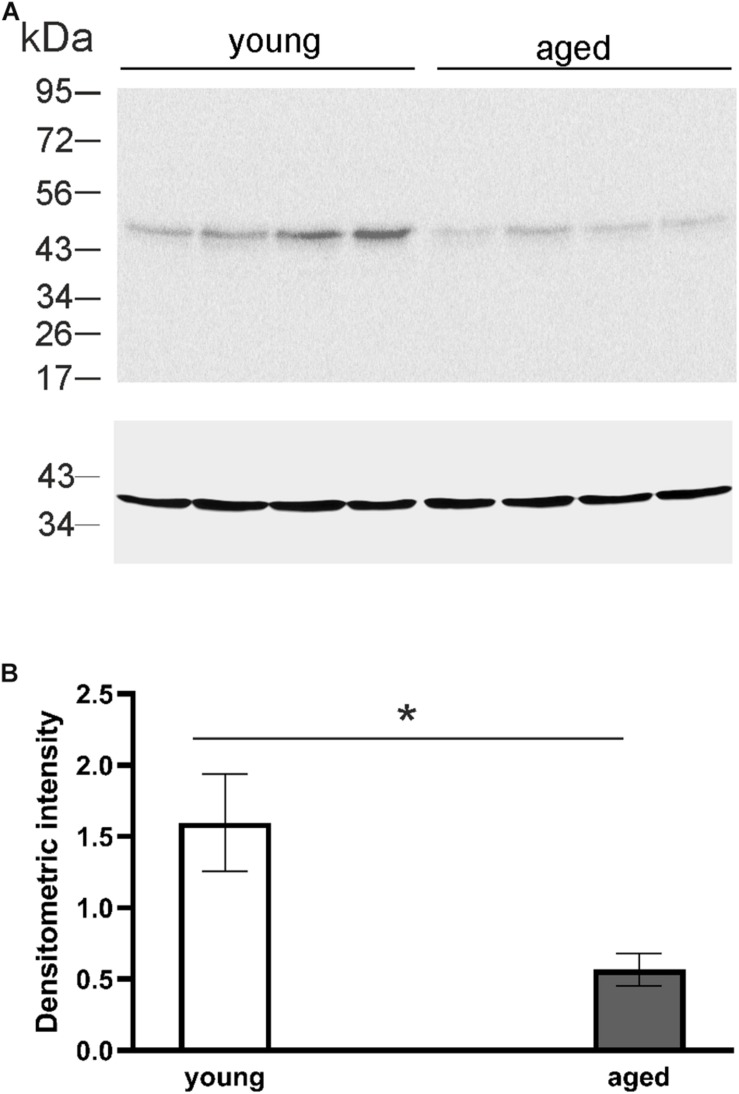
The level of DCX protein in the DG of young and aged opossums. **(A)** Western blot showing labeled bands of DCX and the loading control, GAPDH protein, from four young and four aged brains. **(B)** A quantification of DCX protein levels normalized to GAPDH protein in the DG in young and aged groups. ^∗^*p* < 0.05.

DCX, a marker for migrating progenitor cells and immature neurons, is often used as a marker for adult neurogenesis. To evaluate the differences in adult neurogenesis of young and aged animals, that were used in behavior experiments, Western blot analysis was performed. The DCX level was analyzed densitometrically with GAPDH as a loading control. Examples of Western blot lysates from the hippocampal formation involving mainly DG as illustrated in Figure 5 clearly show that the level of DCX in young opossums was higher than in old animals. Statistical analysis indicated significant differences in DCX protein concentrations between young and old males (Figure 5B).

**FIGURE 6:**
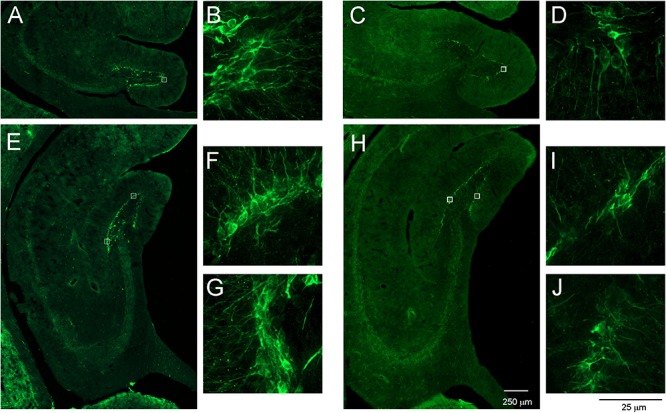
DCX-labeled cells in the DG of young and aged opossums. **(A–D)** Localization of DCX-positive cells and their fibers in the anterior part of the DG. **(B,D)** Higher magnification images from the area marked by box in **(A,B)**, respectively. **(E–J)** Localization of DCX immunopositive cells and their fibers in the posterior part of the DG. **(F,G,I,J)** Higher magnification images from the area marked by box in **(E,H)**, respectively. The scale bar in **(H)** refers to **(A,C)** and **(E,H)**, while the scale bar in **(J)** refers to **(B,D,F,G,I,J)**.

The majority of opossum’s brain was immunofluorescence stained for DCX. The DCX-immunopositive cells appeared in two neurogenic regions, the SVZ and DG of the hippocampal formation (Figure 6). We surmised that the newly proliferated cells in the subgranular layer of the DG migrated short distances and reached the granular layer. As shown in Figure 6, DCX-positive cells were located in the subgranular and granular layers of the DG in both young (Figures 6A,B,E–G) and aged opossums (Figures 6C,D,H–J).

**FIGURE 7:**
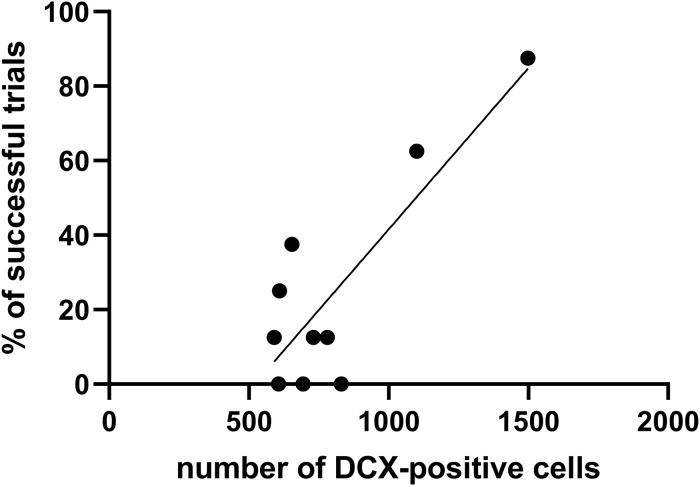
The relationship between the number of DCX-positive cells and percentage of successful trials during the training test. The Pearson correlation test evaluates high correlation (*r* = 0.839, *p* = 0.002) between these two factors.

Furthermore, the number of DCX immunopositive cells was calculated in aged opossums to evaluate the relationships between DCX immunopositive cells and successful trials at days 4 and 5 in the Morris water maze test. A successful trial was determined if the opossum located a hidden platform in the water maze during a 60-s session. The Pearson correlation test showed that correlation occurred between the number of DCX immunopisitive cells and successful trials (Figure 7); where the number of XY pairs was 10, *r* = 0.839, *p* = 0.002.

## Discussion

We examined the association between adult neurogenesis in the DG of young and old opossums and the spatial memory as tested by the Morris water maze test. We found that both young and aged opossums learned to locate the hidden platform in the pool using visual cues. In the aged opossums, the rate of generation of new neurons in DG was significantly lower compared to young animals. However, since neurogenesis still occurred in the DG of older animals, this does not exclude the possibility of adult neurogenesis contributing to memory and learning.

The Morris water maze test is the benchmark test to study spatial learning and memory in rodents, particularly in mice and rats (Morris, 1981). The animal swimming in the pool learns to use the visual cues in the experimental room to locate the hidden platform. This test was used by Kimble and Whishaw (1994) to assess the behavior of opossums, *M. domestica* and reported that the opossums were unable to find the platform.

In our study, however, we modified this test, taking into account that opossums have lower body temperature and metabolic rate than rodents, and quickly develop hypothermia in temperatures appropriate for rodents. Their preference for higher temperature is indicated by the fact that one of the most important conditions for their breeding is high room temperature (26–28°C). We observed that opossums behave very differently in warmer water. When the water temperature in the pool was 26–27°C, opossums learned to find the hidden platform in the water maze test, as opposed to that described in Kimble and Whishaw (1994). The water temperature in their experiments was 21°C, which could have been excessively cold for opossums. Another modification in our experiments was fixing the nest-house on the edge of the pool. Since opossums live a solitary life from the age of 4 months, each of the opossums was kept in a separate cage equipped with a plastic nest-house. Opossums spend a lot of time in their nest-house and are familiar with its appearance. The attached nest-house provided an additional visual cue and encouraged opossums to swim toward the platform. Additionally, the opossum was placed on the platform for 60 s before the probe trial to demonstrate that the environment had not changed. This did not affect the behavior of opossums, because both young and aged opossums that learned to locate the hidden platform during the probe test differed from the opossums that were unable to find the hidden platform. It appears that a combination of these modifications could have motivated the opossums to learn the location of the platform.

We analyzed the behavior of adult opossums in the water maze during aging. Opossums mature sexually by the age of 6 to 7 months and their average lifespan is about 2.5 years. To address whether the cognitive status of animals was associated with neurogenesis, two groups were chosen; young, 6 months old opossums (developed sexually) and aged, about 2 years old opossums. Our results indicate that both groups learned to locate the hidden platform in the pool. However, the main differences between them were the velocity of swimming and thigmotaxis. Young opossums swam faster than aged animals. The reason for this could be body weight, since aged opossums are heavier. The aged opossums displayed more thigmotactic responses, since they spent more time swimming close to the pool wall than young opossums. Thigmotaxis is considered as stress responses in rodents (Treit and Fundytus, 1988; Higaki et al., 2018), but we revealed the second aspect of this behavior in the opossum. Opossums made efforts to climb the pool wall to find a way out. Aged opossums exhibited increased thigmotaxis behavior during the probe test which took more than 50% time in the pool. Additionally, behavioral analysis of the opossums in the pool indicated that they do not like swimming. Given that thigmotaxis involved the opossum climbing the pool wall and trying to get out of the pool, it appeared that aged opossums spent more time figuring out the possibility of getting out of the pool. Most likely, by increasing the number of training sessions we could reduce thigmotaxis behavior in aged opossums.

In the adult brain of mammalian species, including the laboratory rodents proliferation of newly born neurons occurs in two neurogenic structures, the SVZ and DG (for review, see Augusto-Oliveira et al., 2019). In our previous paper we showed that in the opossums cells continuously generate in the SVZ, migrate, and finally reside in the OB as the granule or periglomerular cells and the second neurogenic area is the DG (Grabiec et al., 2009). Here, using DCX as a marker for immature neurons, we found that in the adult DG progenitor cells after divisions generate immature neurons, expressing DCX. Immature neurons develop and grow becoming mature granular neurons and their numbers decline in aged opossums. We demonstrated a correlation between high numbers of DCX cells in the DG of aged opossums and memory performances in the water maze indicating that newborn neurons are required for learning and memory.

Adult hippocampal neurogenesis is frequently associated with learning and memory (Gould et al., 1999; Kempermann, 2002; Deng et al., 2010; Shevtsova et al., 2017), although many studies suggest that a correlation between them is indirect. For example, it has been reported that opioids affect learning (Spain and Newsom, 1991; Miladi Gorji et al., 2008) or that opioids reduce the number of newly born cells in the DG (Eisch et al., 2000; Arguello et al., 2009), demonstrating that inhibition of adult neurogenesis correlates with learning and memory impairment.

Aging also affects learning and memory, and adult neurogenesis reduces with age and sometimes even stops during aging (Kuhn et al., 1996; Bizon and Gallagher, 2003). Drapeau et al. (2003) divided aged rats into two groups: those with impaired and those with unimpaired spatial memory, based on the results from the water maze test. Interestingly, the group of aged rats with unimpaired spatial memory had more newly born neurons in the DG than aged rats with impaired memory. They suggest that a certain threshold number of new neurons in the DG is required for learning, and that cognitive impairment starts manifesting, when the number of newly generated neurons drops below this threshold. In fact, young opossums learned to locate the hidden platform. Therefore, in accordance with Drapeau et al. (2003) we postulate that a critical number of new neurons generated in the DG of opossums is necessary for effective memory formation. This data supports the hypothesis that memory impairments are correlated with the low number of newly born neurons in the DG. However, there are also controversial data that show no correlation between hippocampal neurogenesis and learning and memory (Merrill et al., 2003). In part, these controversies may be due to BrdU administration. It has been reported that increased adult neurogenesis occurred when BrdU was injected 6 days earlier before performing the water maze test (Epp et al., 2007). This is a developmental stage that corresponds to the period of axon growth by newly generated granule cells in the adult DG (Hastings and Gould, 1999).

In line with this, factors that lead to cognitive decline in young animals, such as prenatal stress, also decrease the number of adult-born granular cells of the DG (Lemaire et al., 2000). However, some reports provide evidence against the correlation between memory and the number of the newborn neurons (Van der Borght et al., 2005; Leuner et al., 2006). On the other hand, inhibition of adult hippocampal neurogenesis produces impairments in spatial long-term memory (Snyder et al., 2005).

## Conclusion

In conclusion, aged opossums with better performances in the water maze task had adequately high numbers of newly proliferated cells in the DG, therefore newly born neurons are required for learning and memory.

## Data Availability Statement

All datasets generated for this study are included in the article/supplementary material.

## Ethics Statement

The animal study was reviewed and approved by the First Warsaw Local Ethics Committee for Animal Experimentation.

## Author Contributions

BT, AA, and LG performed the experiments. KB and KT supplied the acquisition of data and were responsible for the manuscript intellectual content. RD contributed to the concept and study, design, and the writing of the manuscript.

## Conflict of Interest

The authors declare that the research was conducted in the absence of any commercial or financial relationships that could be construed as a potential conflict of interest.
